# Evolutionary Dynamics of Strategic Behavior in a Collective-Risk Dilemma

**DOI:** 10.1371/journal.pcbi.1002652

**Published:** 2012-08-23

**Authors:** Maria Abou Chakra, Arne Traulsen

**Affiliations:** Evolutionary Theory Group, Max-Planck-Institute for Evolutionary Biology, Plön, Germany; Harvard University, United States of America

## Abstract

A collective-risk social dilemma arises when a group must cooperate to reach a common target in order to avoid the risk of collective loss while each individual is tempted to free-ride on the contributions of others. In contrast to the prisoners' dilemma or public goods games, the collective-risk dilemma encompasses the risk that all individuals lose everything. These characteristics have potential relevance for dangerous climate change and other risky social dilemmas. Cooperation is costly to the individual and it only benefits all individuals if the common target is reached. An individual thus invests without guarantee that the investment is worthwhile for anyone. If there are several subsequent stages of investment, it is not clear when individuals should contribute. For example, they could invest early, thereby signaling their willingness to cooperate in the future, constantly invest their fair share, or wait and compensate missing contributions. To investigate the strategic behavior in such situations, we have simulated the evolutionary dynamics of such collective-risk dilemmas in a finite population. Contributions depend individually on the stage of the game and on the sum of contributions made so far. Every individual takes part in many games and successful behaviors spread in the population. It turns out that constant contributors, such as constant fair sharers, quickly lose out against those who initially do not contribute, but compensate this in later stages of the game. In particular for high risks, such late contributors are favored.

## Author Summary

The evolution of cooperation is a fascinating topic with a wide range of applications, from microbial evolution to global cooperation of humans in the context of climate change. Motivated by the prospect of dangerous climate change, behavioral experiments of a ‘collective-risk dilemma’ were conducted, where cooperation is in vain unless a threshold is met. This game requires multilateral efforts over several rounds in order to reach a known target and avoid collective loss. We have conducted large scale computer simulations to explore the evolutionary dynamics of strategic behavior in such collective-risk dilemmas. Individuals can react to the contributions of their co-players over the course of the game and adopt their own contributions. The timing of contributions to the public good is a very important issue for long-term problems such as climate change. In this context, it is imperative to know when individuals (or countries) would naturally contribute. We show that a specific behavior, late contributions, is favored, especially when risk is high. Collective-risk dilemmas can by their very nature lead to a detrimental outcome for all involved, and, thus it is crucial to understand the behavior that is expected in such a situation.

## Introduction

Cooperation, between selfish individuals in public goods games [1]–[8], becomes particularly challenging when the prospects are uncertain and a critical number of cooperative acts is required. Investing in the prevention of climate change is in vain if too many other do not invest [9]–[13]. In this context, it may not only be important if we cooperate at all, but also when we cooperate. Motivated by the prospect of dangerous climate change, Milinski et al. have conducted a behavioral experiment to address such a situation [9]. The experiments were designed to capture a collective-risk social dilemma which arises when a group of individuals must cooperate to reach a common target in order to avoid the risk of collective loss. Subjects were distributed into groups of six players and given an initial endowment of 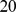 money units (in their case, each unit was worth 2 €). Over 10 rounds, each player could invest 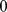, 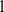 or 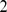 units into a common account. Preceding each investment decision, players were informed about the individual contributions in the previous round. At the end of the game subjects were allowed to keep their savings only if the common account contained at least half of the total endowment of the group; otherwise, all members lost all their savings with a certain risk probability. Milinski et al. found that when this risk is high, contributions increased overall. However, the majority of groups missed the target by a small margin, which is the worst possible outcome; investing nothing would lead to a higher expected payoff.

The experiment of Milinski et al. has triggered numerous theoretical investigations [14]–[17]. The focus has been to use an evolutionary game in order to analyze the consequences of a target threshold, which represents a serious complication over the usual public goods games [8], [17]–[21]. However, these studies considered only two behaviors, cooperation or defection, and assumed that individuals do not react to the contributions of their co-players over the course of the game. This means that effectively in these previous investigations the game was limited to a single round even though the full game consists of multiple rounds. However, a direct influence of co-players on individuals' decision emerges when there are several subsequent stages [22]–[24] of investment and it is not clear whether individuals should contribute in early or in late stages of the game. Herein, we explore the evolutionary dynamics of strategic behavior in such multi-round game by analyzing the timing of the contributions. With this method, we aim to understand the natural behavior in such kind of situations. This behavior is of particular relevance in the context of dangerous climate change, which has been modelled as a collective-risk dilemma [9]. Should we be pessimistic towards the prevention method used for climate change, especially when major industrial nations fail to fulfill their targets in CO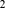 reduction in time? Or is this a natural behavior in such collective-risk dilemmas? Under which circumstances would early contributions be favored? In order to investigate strategic behavior in this game, we explore the general characteristics of such behavior through large scale computer simulations. We use evolutionary game dynamics [25]–[27] to infer which strategies are particularly stable in collective-risk games.

## Results

### Evolutionary Game Dynamics in the Collective-Risk Dilemma

We employed an evolutionary game, in which success is measured by the average payoff over many collective-risk dilemmas. Such a collective-risk game is played among 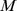 individuals selected at random from a well mixed population of size 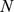. An individual player commences each game with an initial endowment of 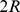, where 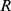 is the total number of rounds played in a game. In each round 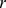, players simultaneously invest 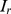 units into a common pool. The total investment of a player is 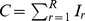. In our analysis, we focused on a six player game in which players can invest 0, 1 or 2 units for ten rounds, as in [9]. We also discuss the consequences of relaxing these assumptions.

The whole group collectively has to invest a target sum 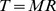 by the end of the game after 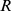 rounds. If they succeed, they can keep what they have not invested. If they fail, they lose what they have not invested with probability 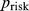 and keep it with probability 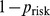. Thus, a player obtains a payoff of 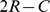 when the target is reached and an average payoff 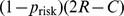 when the target is missed. Note that the individual payoff is independent of the timing of the contributions – but this timing can be crucial for the interactions among the players.

This collective-risk dilemma has a large strategy space and a large set of Nash equilibria. Each situation in which the group of players collectively contributes exactly 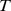 and no player invests more than 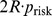 is a Nash equilibrium, irrespective of the distribution of contributions within the group. For example, for 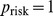, half of the players could invest 2 units in each round and half of them nothing is a Nash equilibrium, despite being unfair. In this situation, the target is exactly met. If those who invest 2 units would invest less, the target would not be met. If those who invest nothing start contributing, these contributions would be in vain. In general, such deviations from the Nash equilibrium are disadvantageous for the individual in high risk situations. In addition, the situation in which no one contributes is a Nash equilibrium, because it takes more than one player to reach the target.

A behavior can be defined from the individual contributions over the 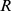 rounds. In our case, each player can choose between three actions in each round, thus there are 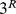 different behaviors, increasing exponentially with 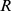. If behaviors are independent of the actions of others, we can collapse the whole dynamics into a single round game and identify strategies such as defectors (someone who does not contribute, 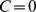), fair sharers (contributing half of the endowment, 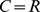), altruists (contributing everything they have, 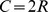), or many others. However, when behaviors also depend on the actions of the other 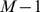 players, identifying the underlying strategies becomes much more challenging. In our case, the 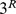 different behaviors are only based on the total amount that has been invested so far, a reasonable assumption in a context where it is difficult to monitor individual actions. Nevertheless, this assumption can lead to complex strategies and behaviors. A player's strategy determines how much to contribute in a given round, depending on the collective contributions so far. We assume that players invest more (or less) once the collective contributions have reached a certain amount 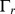. A player could aim to invest less when contributions are high, but it may also be reasonable to compensate the missing contributions of others. We defined a player's strategy based on a threshold and the contributions when the invested sum so far is above or below this threshold. For instance, a player could invest 2 in round 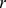 if the total investment so far is above his threshold value and 1 otherwise. The contributions and thresholds can be different for each round, see Methods for a concrete example. This combination produces a large strategy space. Note that, an individual with a specific strategy (defined by the thresholds and contributions) can show a wide range of behaviors based on the common pool and hence on the strategies of other co-players.

**Figure 1:**
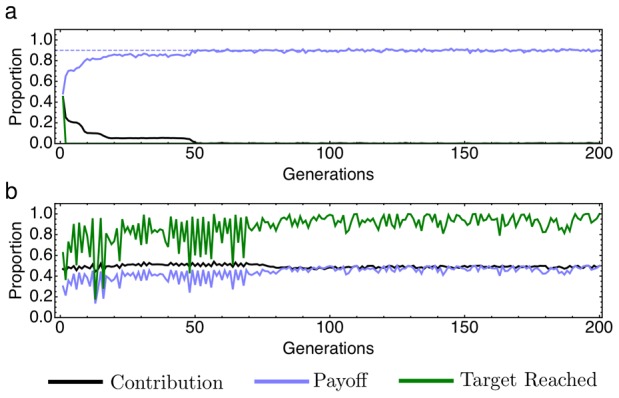
Sample trajectories for the evolutionary dynamics in collective-risk dilemmas. (a) For small risk probability, 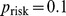, the initially random contributions quickly drop to zero. Consequently, the target is never met and the average payoff is 90% of the initial endowment, as expected. (b) For high risk probability, 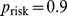, strategies investing the target sum are favored. After a few generations, in the majority of games the target is met, leading to an average payoff of almost half of the initial endowment (parameters 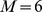, 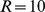, 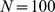, 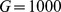, 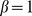, 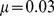, 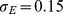).

In evolutionary game dynamics, the payoff determines the fitness and thus more successful strategies spread in the population. In our setup, ‘evolution’ operates at the level of strategies while ‘selection’ operates at the behavioral level. Evolutionary game dynamics were simulated using a mutation-selection process in a population of finite size [27], cf. Methods. The evolutionary game dynamics of strategic behavior depends crucially on the risk probability 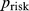. As an illustration, Fig. 1 shows typical simulations for low risk (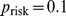) and high risk (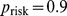), the parameter values analyzed in a behavioral experiment with students by Milinski et al. [9]. Within the first 200 generations, the average contributions and the average payoff values stabilize. As expected, for 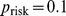 individuals do not contribute and the average payoff is 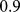 of the initial endowment, cf. Fig. 1a. In contrast, for 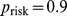, individuals on average contribute half of their endowment (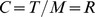), cf. Fig. 1b. In this case, the target is reached with a probability larger than 80%, leading to an average payoff substantially larger than 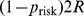. Note that the average payoff when the target is met, 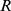, is identical to the average payoff with zero contributions for 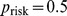.

**Figure 2:**
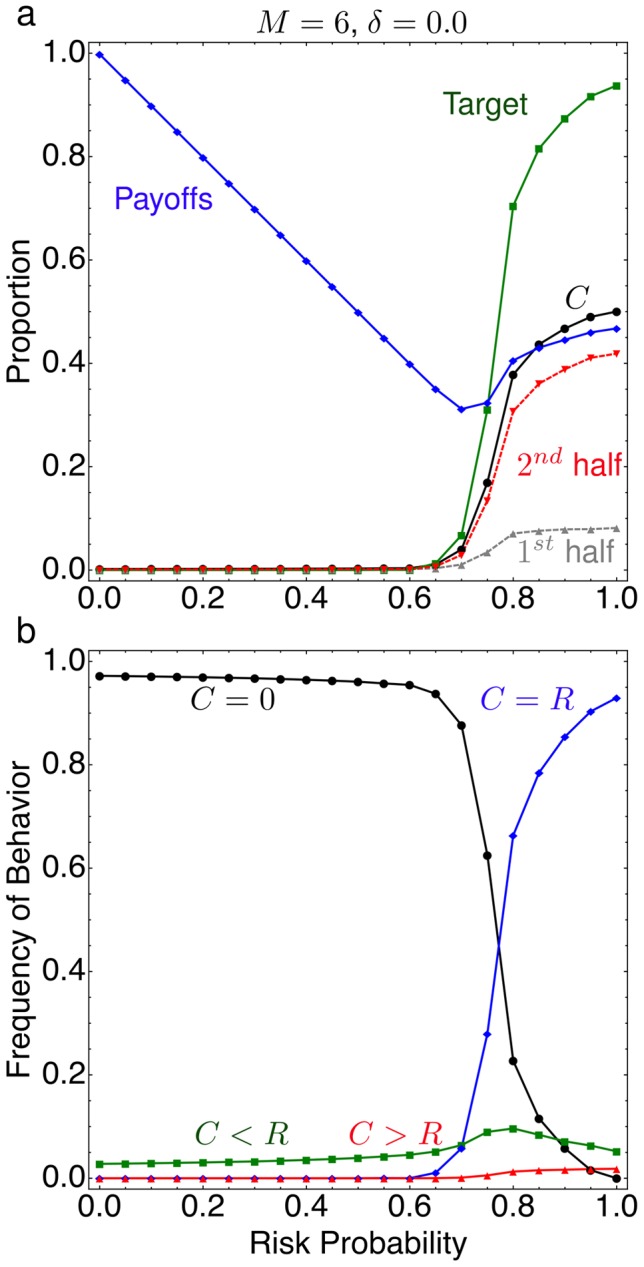
Summary of the evolutionary dynamics in collective-risk dilemmas. (a) The probability to meet the target investment, average payoff, total investment and investment in the first and second half of the game for different risk probabilities 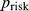 (all payoffs and investments are measured in proportions of the total endowment). Players do not invest for 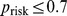, for 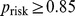, players invest up to half of their endowment, and at 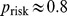, more than half of the games meet the target. Investment mainly occur in the second half of the game. (b)The total investments in the game, 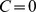 behavior occurs at high frequencies for 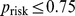, while the 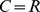 behavior dominates for 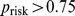. Behaviors where 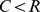 occur for all 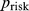 at low frequencies, while over-contributors, 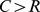, are also rare but only seen for very large 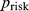 (averages over 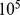 generations from 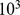 independent realizations parameters 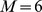, 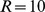, 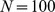, 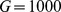, 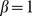, 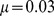, 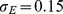).

When 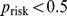, it is not worthwhile to contribute to the common account, because the expected payoff for not reaching the target is still higher than the payoff when the target is met and everyone contributes half of their endowment, 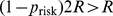. We find that, simulations for risk values up to 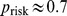 lead to an average payoff of (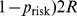. Our simulations show that for 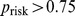 the average payoff increases to values close to half of the initial endowment, which would be the optimal solution for high risks. This happens when the probability to meet the target reaches values much larger than 50%, see Fig. 2a.

The probability to reach the target decays when there are more errors in strategy inheritance - they lead to changes in the contribution patterns which make it more difficult to evolve a solution for the game. We incorporate errors in our evolutionary process with probability 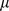, cf. Methods. Consequently, the average payoff decreases with increasing error probability 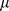. The diversity also increases for smaller intensity of selection 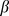. Increasing 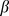 stabilizes the population faster and quenches the overall effects of 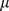.

### Behavioral Analysis

The dynamics of strategies can also be addressed on the behavioral level, which reflects the interaction of players and the corresponding strategic aspects. We use the contributions of individuals to differentiate between the different behaviors for games under various 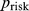. A behavior with 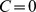 represents the classical defector, such players would always invest 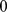. E.g. in a four round game investing 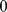 in each round, they would have 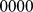 as the corresponding behavior. The opposite behavior, an unconditional altruist, is represented by 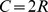, which means the player contributes 2 in each round, e.g. 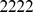 in a four round game. A behavior with a 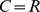 represents any behavior where a player contributes half of the endowment over the rounds; there are many corresponding behaviors, e.g. in a four round game such a player could contribute a total of 4 units in 19 different patterns, 1 in each round (1111), 1 in two rounds and 2 one round (i.e. 0112, 0121, 0211, 1012, 1021, 1102, 1120, 1201, 1210, 2011, 2101, 2110), or 0 in half of the rounds (i.e. 0022, 0202, 0220, 2002, 2020, 2200). In general, there are 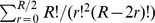 behaviors with 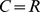, increasing rapidly with 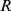. Note that each of them – and any mixture of them – is a Nash equilibrium. For an efficient analysis we divided strategies into four behavioral categories, 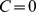, 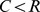, 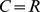, and 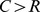, see Fig. 2b. The 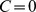 behavior occurs at high frequencies for 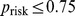, while the 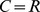 behavior dominates for 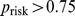. Behaviors where 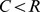 occur for all 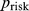 at low frequencies, while over-contributors, 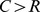, are also rare but only seen for very large 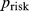. There is a single behavior associated with 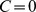, however, there are many behaviors with 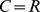. The increase in frequency of 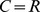 when 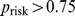 could be attributed to any of them. Therefore, we divided the game into two halves and analyzed the contributions. It turns out that at least 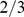 of the total contributions are made in the second half of the game for 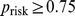, Fig. 2a.

Next, let us infer which behaviors are responsible for meeting the target when the risk is high. Interestingly, a single behavior dominates for all 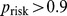 which can be described as a ‘fair rational’ behavior. The name indicates that these players invest their fair share 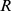, but also employ a reasoning related to backward induction for the strategic timing of their contributions. In this case, half of the endowment is contributed in the second half of the game and nothing is contributed in the first half of the game (e.g. in a game with 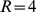 the dominating behavior can be represented as 0022). Such behavior is consistent with the contribution increase observed in the second half of the game, Fig. 2a. We find this for different round numbers ranging from 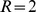 to 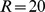, and a wide range of the other parameters (see Fig. S1). For instance, if we vary the maximum contribution permitted in each round, the same ‘fair rational’ behavior emerges, with contributions starting as late as possible (see Fig. S2). This indicates that the ‘fair rational’ behavior is preferred in such collective risk game when risk is high.

**Figure 3:**
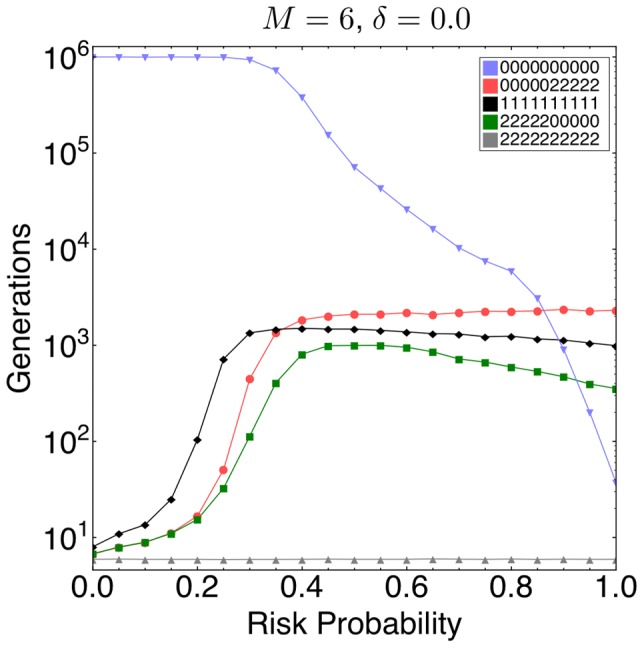
Stability of behaviors in a collective-risk dilemma. Simulations were conducted with the following five behaviors from a 10 round game: 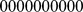, 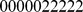, 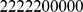, 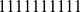, and 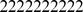. As expected, 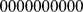 was most stable for all 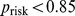, indicating that it is difficult to evolve cooperation in this game. For 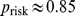 both 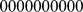 and 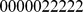 were equivalently stable. As 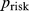 increases the stability of the 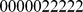, ‘fair rational’ behavior, improves (Averages from 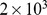 realizations, parameters 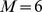, 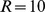, 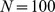, 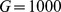, 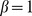, 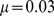, 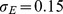).

We assessed the robustness of such behavior by initializing a homogenous population and analyzing the duration for which the behavior is maintained at a frequency greater than half of the initial population size. In Fig. 3, we analyze five different behaviors: Non-contributors with 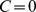, e.g. a 4 round game would have 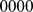 as the corresponding behavior, altruists with 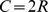, i.e. 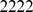, and three behaviors with 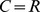: (i) the ‘fair rational’, i.e. 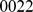, (ii) fair naive, i.e. 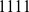, and (iii) the reverse of the ‘fair rational’, i.e. . Simulations show that as 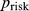 increases the stability of the ‘fair rational’ behavior improves. 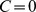 was most stable for all 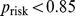. For 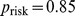 the stability of the 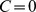 behavior was similar to the ‘fair rational’. When 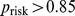 the ‘fair rational’ behavior is more stable than all other behaviors including the defecting 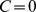 behavior. The stability of the ‘fair rational’ behavior indicates that later contributions are favored for high risk, in line with our simulations of the mutation-selection balance.

### Model Exploration

Our approach allows us to explore the impact of several aspects that have not yet been analyzed in a behavioral experiment. For a comprehensive analysis, we considered the effects of group size, interest on the common account, uncertainty in target, and continuously decreasing risk curves. First, we explored the impact of group size in such collective-risk game.

**Figure 4:**
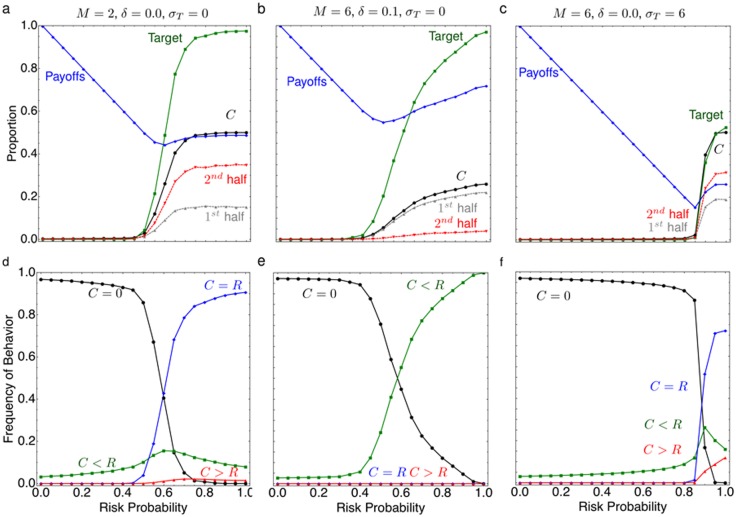
Variations of the collective-risk dilemma. Top: the probability to meet the target investment, average payoff, total investment and investment in the first and second half of the game. With smaller group size (a) and interest (c), contributions started at 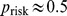. With interest, investments mainly occur in the first half of the game. If the target varies, they only start for 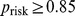 (e) and the success frequency decreases. Bottom: the total investments in the game (all payoffs and investments are measured in proportions of the total endowment). In general the 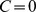 behavior occurs at high frequencies for low 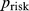. (b) For small groups, the 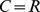 behavior dominates for 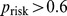. (d) With interest, the 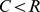 behavior dominates for 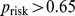. (f) If the target is uncertain, the 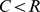 behavior dominates for 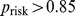 and 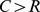 occurs for very large 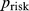 (averages over 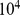 generations from 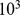 independent samples, parameters were 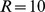, 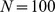, 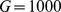, 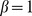, 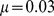, 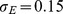, otherwise stated in the figure).

When only few players have to coordinate their actions, a smaller strategy space has to be explored. In a game with 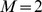, players do not invest for 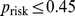, for 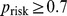, players invest up to half of their endowment and at 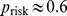, more than half of the games meet the target. Investment still mainly occur in the second half of the game; 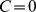 behavior occurs at high frequencies for 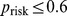, while the 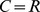 behavior dominates for 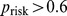. Behaviors where 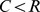 occur for all 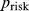 at low frequencies, while over-contributors, 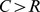, are also rare but again only seen for very large 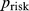, see Fig. 4a–b. Furthermore, simulations show that when players are in smaller groups, contributions start at a lower risk value, compared to larger groups. For instance, for 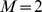, contributions started for 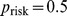 (Fig. 4a), for 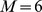 contributions start at at 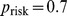 (Fig. 2a), and for 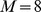, contributions started for 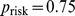 (Fig. S1a–d). Consequently, the payoffs increase to values above 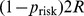 only for higher risk probability in larger groups.

Second, we added an interest on the common account, such that early investments are more valuable. This only has an impact if the interest is high enough to replace a late contribution by a smaller, earlier contribution. For instance, simulations show that contributions begin to increase when 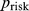 increases above 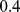, when there is an interest of 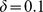 on the common account. When 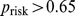, the target is met with probability larger than 50%, in this case cf. Fig. 4c. We also observed that the 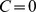 behavior occurs at high frequencies for 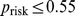, while the 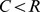 behavior dominates for 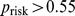, see Fig. 4d. It is now possible to reach the target with such behavior. However, behaviors where 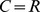 occur for all 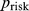 at low frequencies. It turns out that (unlike in the simulations without interest) contributions are made in the first half of the game, Fig. 4c, this was consistent for different group sizes, cf. Fig. S1e–h. Interest also substantially increases the noise in the system; when interest was added to the common account individuals had an incentive to contribute early, however as a result invaders infiltrated and disrupted the stable equilibrium.

Finally, we considered the effects of uncertainty in the target and smooth risk curves. If the target is not exactly known, it is substantially more difficult to evolve cooperation. Adding noise to the target causes the contributions to start at higher risks, but also causes a drastic decrease in the probability that the target reached. For example, without such noise and 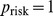 the target was reached with a 95% probability. But for a target subject to Gaussian noise with standard deviation of 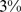 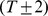, the target was reached with only 80% probability, this was consistent for different group sizes, cf. Fig. S1i–l. Failure rate increased with increasing uncertainty in the target, for instance a target subject to Gaussian noise with standard deviation of 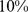 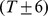 dropped success to a 50% probability, Fig. 4e. Such uncertainty caused a change in behaviors, this is observed by a frequency increase in overcontributors (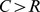) and noncontributors (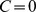) and a decrease in the fair sharers (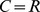), Fig. 4f. Despite the increase in failure probability, contributions reached half of the endowment when 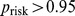, Fig. 4e.

We also considered a risk curve that is smooth instead of the step function, such that higher contributions continuously decrease the risk. Also in this case, the general picture does not change - late contributions are favored for sufficiently high risk.

## Discussion

The collective-risk dilemma is characterized by thresholds which capture risky collective-actions. Due to its potential relevance for dangerous climate change and other global crisis or risky social dilemmas, the general characteristics underlying such game structure are of crucial interest.

Our model captures strategic elements in collective risk dilemmas by allowing individuals to interact and influence each other. We extracted a robust natural behavior for different risk levels. Our simulations of the collective-risk game unveiled a high abundance of a ‘fair rational’ strategy, such that the fair share is relinquished as late as possible. We vary the maximum contribution allowed, interest and uncertainty and analyze how all these factors influence the timing of contributions. We show that maximum payment dictates when contributions commence. Players procrastinate their contributions as much as possible. This implies that the maximum contribution possible (or allowed) per round determines the timing of contributions. Additionally, we show that interest to the common account can also affect the timing of contributions–individuals had an incentive to contribute early. This suggests that for time sensitive collective actions, incentives can be used to induce earlier contributions. We also show that uncertainty can cause a lack of coordination; simulations resulted in a decrease in success when the target was uncertain. Failure can also arise from an increase in group size or a decrease in risk probability. For larger group size the probability for successful cooperation decreases, as an individual's probability of being pivotal declines. However, simulations show that increasing risk probability quenched some of these uncertainties, and in turn, contributions increased. This suggests that chances of success increase when all the uncertainties are resolved. Moreover, it is essential to be informed about the maximum possible contribution, otherwise one may be too optimistic about the possibility to compensate in later stages. Finally, to understand the differences between the sequential game where individuals play in sequence [22], [28]–[35] and the collective-risk game where individuals play simultaneously in a sequence of rounds [9], [11], [12], we expanded the scope of our computational model by allowing for sequence allotment for individual players, see Fig. S3. Our simulations reveal that a sequential game with 10 players has a lower efficiency in comparison to a 10 round collective-risk game for high risk probabilities.

The collective-risk game requires coordination and, thus, a player needs to rely on deductive reasoning to ensure others are in agreement. A player should not invest if the chances are low that the other group members will invest sufficiently. Thus it is important to anticipate how others will behave. As the group number increases influencing – or predicting – the behavior of co-players becomes exceedingly difficult. However, deductive reasoning shows that the number of possible behaviors differ from round to round. If players wait and the pool remains empty, the number of strategies that allow to meet the target quickly drops. When nothing has been invested in the first half of the game, there is a single fair share behavior remaining, the ‘fair rational’. By simply waiting, players are forced to play the ‘fair rational’ strategy or risk losing everything; such natural enforcement is most effective when risk is high. One rationale for the ‘fair rational’ behavior is that it can induce other players to contribute half of their endowment. Early contributions may seem more intuitive in the face of high risk. But they allow the invasion of other strategies and endanger the success of the game. Numerically, all fair share behaviors have the same payoff, but they diversify when considering invasions by deviating types. However, deviation from the ‘fair rational’ behavior is unforgiving and decreases the fitness of the deviating individuals, either collectively when the target is not met or individually when deviators have contributed too much. The ‘fair rational’ behavior leaves no room for conscious or erroneous deviation, and all co-players must contribute or risk consequences of failure for all.

Players aiming to ‘play it safe’ by overcontributing or contributing early do not necessarily have a positive effect in collective-risk dilemmas, which can by their very nature lead to a detrimental outcome for all players. Thus, it is beneficial to have a strict behavior enforcing others to act alike, especially when stakes are high.

## Methods

### Individual Strategies

Each individual has a strategy composed of a threshold, 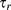 and the contributions above and below the threshold for each round. The investment is thus determined by a player's strategy as well as the collective contributions so far. For instance, a player could invest 2 when the collective contributions are above (or equal to) his threshold 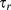 and 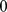 otherwise. We denote such a strategy in round 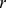 by 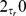. A player that aims to compensate the missing contributions of others could instead have a strategy, such as 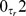 - he would invest if the threshold is not met, but he would stop investing once the common pool is sufficiently filled.

As an example, consider a game with two players and two rounds, 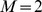 and 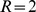. The strategy of player one is 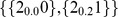. Player two has strategy 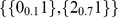. Since the common pool is empty in round 1, 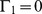, we have 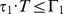 for player one, who thus invests 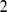. For player two, we have 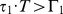, which leads to an investment of 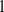. Now, in round 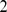 we start with a common pool 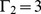. Consequently, for player one 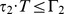 – an investment of 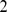. Also for player two, we have 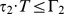 – an investment of 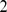. As a result, the total investment after two rounds is 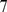 and the target 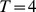 is met. Thus, player one obtains a payoff of 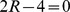 and player two 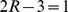. Since payoff determines fitness, this means that the strategy of player two tends to spread.

At the beginning of our simulations, all individuals have different random strategies, i.e. all contributions are 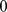, 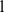, or 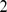 with the same probability and all thresholds are uniformly distributed between 0 and 1.

### Evolutionary Game

In one generation, 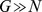 such game are played, such that an individual on average plays 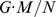 games. The individual's payoff, 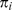, is calculated as the average payoff of all games played. At the end of a generation, the payoff is translated into a fitness value 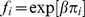, where 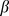 measures the intensity of selection [36], [37]. Higher payoffs increase an individual's reproductive potential towards the next generation.

The next generation is selected using the Wright-Fisher process where the individual's fitness is used to weigh the probability of choosing an individual for the new population [38]–[40]. Offspring inherits the strategy of the parent at the end of a generation (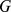 games). We also incorporate errors in this process. Errors occur with a probability 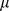 for the thresholds 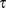 and the investments of each round independently. If they occur, errors in the threshold values add Gaussian noise with standard deviation 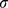 to them. If an error in a contribution occurs, a random contribution is chosen, e.g. in the example above, 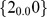 in round 1 could be replaced by 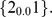 Once a new population is selected the process is repeated for multiple generations and the average of the dynamics is analyzed.

### Stability Simulations

To explore the stability of the different behaviors, a homogenous population was initiated using the same strategy for all individuals. In the simulation the population evolved under selection and mutation parameters. For each strategy we calculated the duration when the frequency dropped below half of initial population size, since this is a natural requirement for another strategy to take over (all our strategies can be invaded in a finite population by neutral drift. So eventually, any strategy will be replaced due to mutation, selection and drift). Averages were computed over 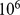 generations from 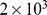 realizations.

### Simulation Details

Our simulations are written in C++ and were run on a 240 core Linux cluster. The computer code is available upon request.

## Supporting Information

Figure S1
**Variations of the collective-risk dilemma.** The panel in grey (g) is our default parameter choice, based on Ref. [3]. The probability to meet the target investment, the average payoff, the total investment and the investment in the first and second half of the game are shown for different risk probabilities 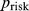 (all payoffs and investments are measured in proportions of the total endowment). In this figure, we explore the interplay of group size with interest and uncertainty in the target. Simulations show that with larger group size contributions start at a higher risk value, compared to smaller groups. Adding interest 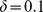 caused the contributions to switch to the first half of the game, in contrast to all other variations we have analyzed. Adding target uncertainty caused the success frequency to decrease (averages over 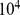 generations from 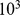 independent realizations; parameters 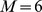, 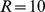, 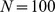, 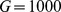, 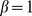, 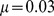, 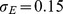, 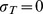, unless otherwise stated in description above).(TIFF)

Figure S2
**Variations of the maximum contribution allowed in a collective-risk game.** Maximum contribution allowed was varied from 1 up to 10. In all cases, contributions start as late as possible. Given a 10 round game and a maximum contribution of 1, players contributed 1 in each round to meet the target, however in a maximum contribution of 5 game, players began contributing the ninth round (averages from independent realizations; parameters 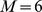, 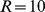, 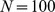, 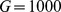, 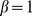, 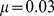, 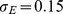, 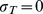).(TIFF)

Figure S3
**Evolutionary dynamics comparison between a collective-risk game and a typical sequential game.** Panels (a) and (b) show the probability to meet the target investment, average payoff, total investment and investment in the first and second half of the game for different risk probabilities 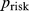 (all payoffs and investments are measured in proportions of the total endowment) for collective risk and sequential games, respectively. (c) The total investments in the collective risk game, 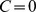 behavior occurs at high frequencies for 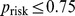, while the 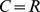 behavior dominates for 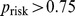. Behaviors where 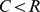 occur for all 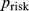 at low frequencies, while over-contributors, 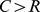, are also rare but only seen for very large 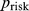 (d) The total investments in the sequential game, 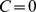 behavior again occurs at high frequencies for 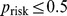, now the 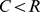 behavior dominates for 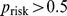. Behaviors where 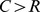 start to increase by 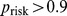 and 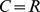 are rare. (averages over 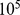 generations independent realizations parameters for collective risk we set 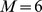 and 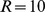, for sequential game we set 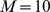 and 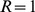 while other parameters remained the same 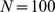, 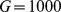, 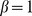, 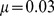, 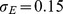, 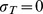).(TIFF)

Text S1 **Supporting information for evolutionary dynamics of strategic behavior in a collective-risk dilemma.** We conduct a comprehensive analysis in which we consider the effects of group size, interest on the common account, uncertainty in target, and continuously decreasing risk curves. Furthermore we analyze the maximum contribution allowed in a collective-risk game and conduct a comparison between a collective-risk game and a typical sequential game.
(TEX)
