# Supplementary figures and images for: Evolutionary Dynamics of Strategic Behavior in a Collective-Risk Dilemma
Source: PLoS Comput Biol. 2012 Aug 23;8(8):e1002652. doi: 10.1371/journal.pcbi.1002652 (PMC3426567; doi:10.1371/journal.pcbi.1002652)

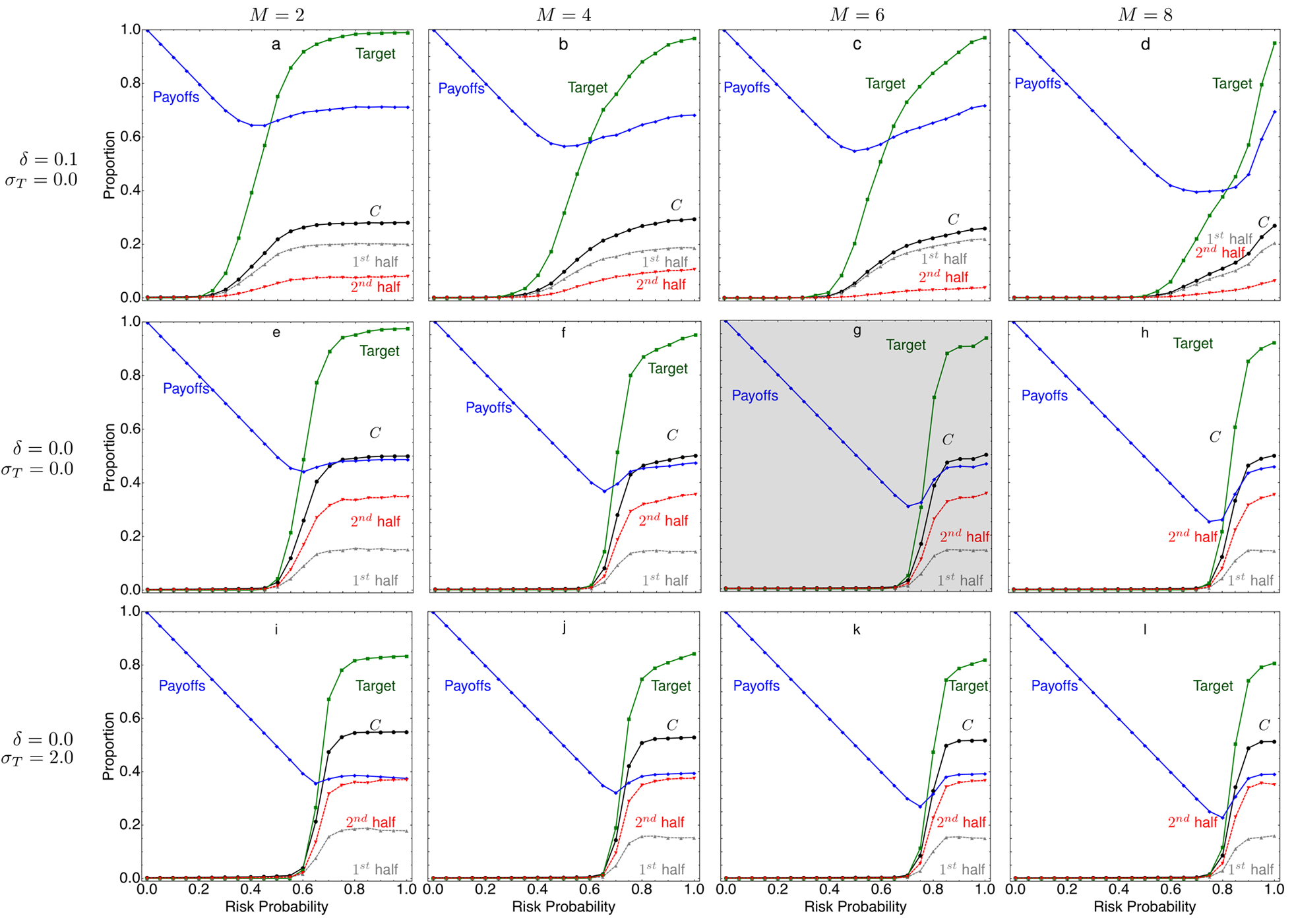

Supplement: Figure S1 — Variations of the collective-risk dilemma. The panel in grey (g) is our default parameter choice, based on Ref. [3]. The probability to meet the target investment, the average payoff, the total investment and the investment in the first and second half of the game are shown for different risk probabilities (all payoffs and investments are measured in proportions of the total endowment). In this figure, we explore the interplay of group size with interest and uncertainty in the target. Simulations show that with larger group size contributions start at a higher risk value, compared to smaller groups. Adding interest caused the contributions to switch to the first half of the game, in contrast to all other variations we have analyzed. Adding target uncertainty caused the success frequency to decrease (averages over generations from independent realizations; parameters , , , , , , , , unless otherwise stated in description above). (TIFF) [file pcbi.1002652.s001.tiff]

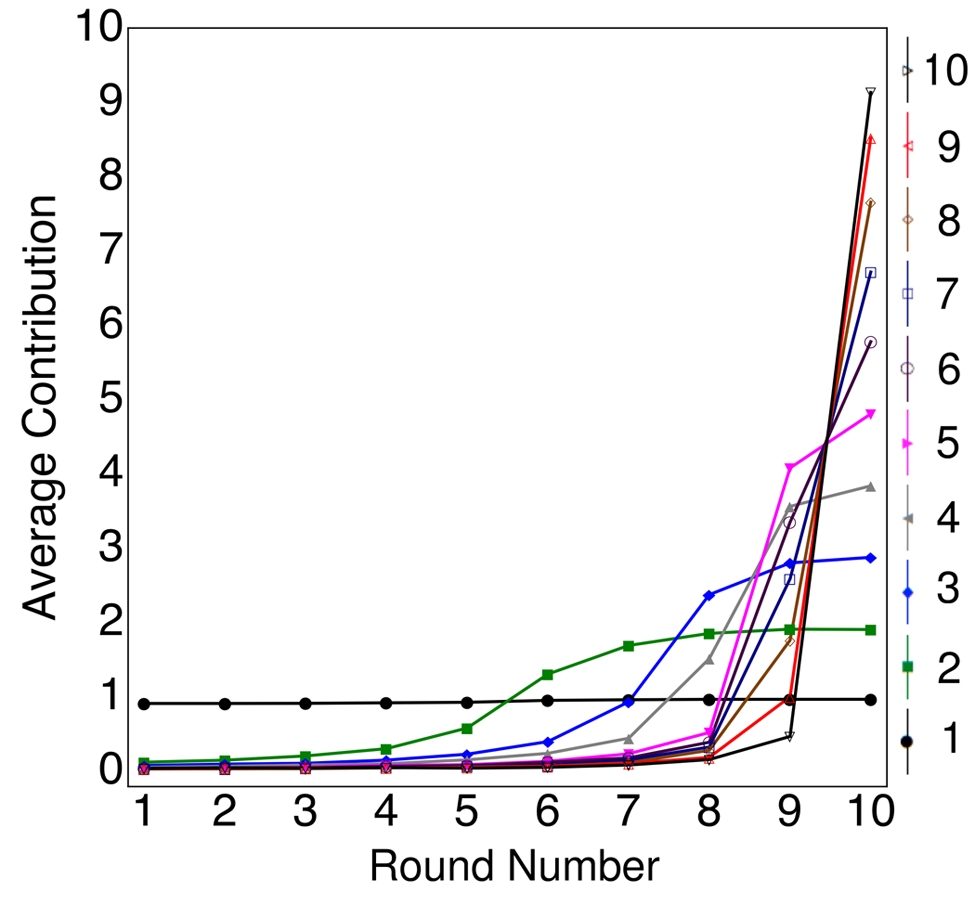

Supplement: Figure S2 — Variations of the maximum contribution allowed in a collective-risk game. Maximum contribution allowed was varied from 1 up to 10. In all cases, contributions start as late as possible. Given a 10 round game and a maximum contribution of 1, players contributed 1 in each round to meet the target, however in a maximum contribution of 5 game, players began contributing the ninth round (averages from independent realizations; parameters , , , , , , , ). (TIFF) [file pcbi.1002652.s002.tiff]

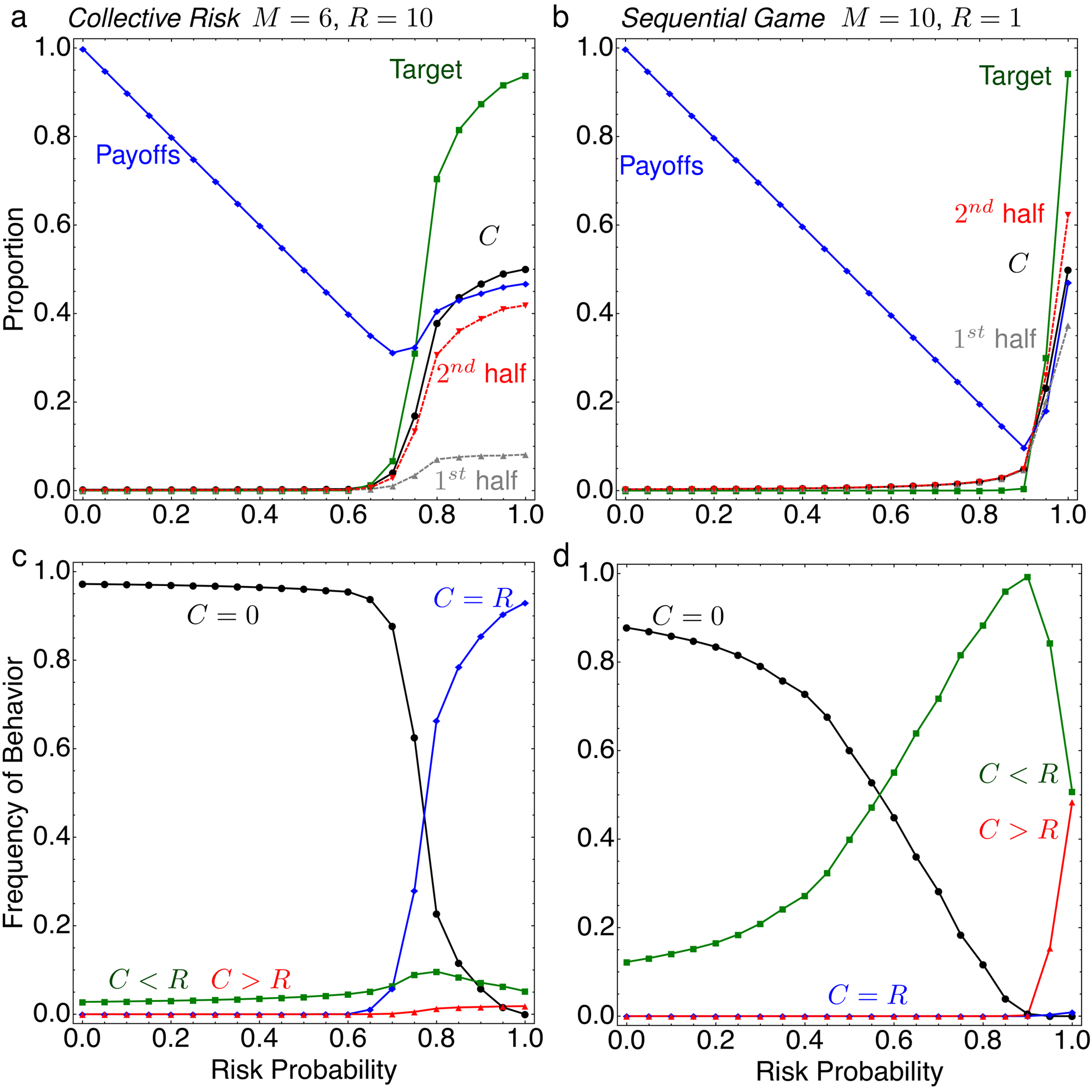

Supplement: Figure S3 — Evolutionary dynamics comparison between a collective-risk game and a typical sequential game. Panels (a) and (b) show the probability to meet the target investment, average payoff, total investment and investment in the first and second half of the game for different risk probabilities (all payoffs and investments are measured in proportions of the total endowment) for collective risk and sequential games, respectively. (c) The total investments in the collective risk game, behavior occurs at high frequencies for , while the behavior dominates for . Behaviors where occur for all at low frequencies, while over-contributors, , are also rare but only seen for very large (d) The total investments in the sequential game, behavior again occurs at high frequencies for , now the behavior dominates for . Behaviors where start to increase by and are rare. (averages over generations independent realizations parameters for collective risk we set and , for sequential game we set and while other parameters remained the same , , , , , ). (TIFF) [file pcbi.1002652.s003.tiff]
